# Skin Diseases and Syphilis

**Published:** 1894-02

**Authors:** 


### *SELECTIONS AND ABSTRACTS.*

---

### SKIN DISEASES AND SYPHILIS.

EDITED BY DR. M. B. HUTCHINS.

### EMPLOYMENT OF SULPHUROUS WATERS IN THE NORMAL TREATMENT OF SYPHILIS.

We have here, as is well known, a practical question of the utmost importance and one much discussed. It is also well known that certain authors do not hesitate to consider treatment by sulphurous waters as dangerous in syphilis. Dr. Dresch, who has just published a short study of the question, thinks he can affirm, in basing his belief on the numerous works of former writers, Colonier, Astrié, Garrigon, etc., and upon his own practice, that the sulphates, and especially the sulphites and hyposulphites which penetrate the blood and the meshes of the tissues, dissolve the albumino-mercurial compounds which fix the salts of mercury in our tissues and thus render their elimination easy. This elimination is also stimulated at the same time by the increased activity impressed upon the cutaneous, urinary and mucous excretions.

The benefits from sulphurous waters in the treatment of syphilis are thus, according to him, manifold. They have, first of all, the advantage of eliminating the mercury accumulated in the organism by the treatment called that of extinction, generally adopted in France according to the principles laid down by Fournier. Now, this mineral poison is not without its serious inconvenience, for the various organs which it impregnates and of which, perhaps, it favors sclerosis as much as the syphilis itself, or the various diatheses which the patient may present.

Furthermore, in causing this mercury in the tissues to move along, it may again enter the circulation and produce upon the economy the same physiological and therapeutic effects as when first administered. That which proves the reality of this extraordinary statement is that sulphur water, taken at the same time as mercury, prevents salivation, and is, on the other hand, capable itself alone to provoke salivation in those who have taken mercury before the thermal cure, and this after a considerable lapse of time from the cessation of all mercurial treatment. Thus, according to the author, should one not carry out a mercurial treatment at sulphur baths in those alone who cannot tolerate mercurial preparations? Otherwise, to give at the same time mercury and sulphurous water constitutes, according to the author, a veritable nonsense, for in organisms capable of supporting mercury the elimination of the medicine is accomplished in their case so extremely fast that the organism is in no wise impregnated. We must, on the contrary, let the sulphurous treatment follow the specific cure. In this way we get out of the mercury all it can give in this coming and going, as it were. We establish in the organism from without inwardly medication by mercury alone, and from within outwardly sulphurous medication alone—medication of elimination. Sulphurous waters can then render immense service in severe and rebellious cases, permitting of a cure by mercury and causing habituation to the drug to cease. When the ground has been in a way renewed by the sulphurous washings a new anti-syphilitic treatment may triumph over accidents which had previously resisted all medication.—*L. Brocq (Paris), in Journal of Cutaneous and Genito-Urinary Diseases, January*, 1894.

---

DYSTROPHIE PAPILLAIRE ET PIGMENTAIRE.—Dr. J. Darier. (*Annales de Dermatologie et de Syphiligraphie*, Tome IV., No. 7, July, 1893, p. 865.)

Two cases are detailed to which M. Darier gives the above name. They are similar to the cases described by MM. Pollitzer and Janovsky under the name Acanthosis nigricans. (The International Atlas of Rare Skin Diseases. Fasc. IV. 1890.)

Both cases occurred in females suffering from abdominal cancer. The condition is one of deep pigmentation of the skin, brown in color, but in some places almost black; this is followed by great hypertrophy of the papillæ. It commences insidiously, but may become very generally developed in the course of a few months. The principal seats of election are the neck, especially about the nape, and to a lesser degree the flexures, though it may also occur on the hands, round the umbilicus and around the mucous orifices of the face, as in one of these cases. A full account of the histological characters is given, and the details differ in some respects in sections from various situations, but in all there is an enormous thickening of the horny layers, and a more moderate increase in the thickness of the malphigian and granular layers, the sweat glands being quite normal.

Attention is called to the differential diagnosis between this affection and the bronzing of Addison's disease, ichthyosis, and senile or seborrheic warts; on the other hand, there are certain points of resemblance between this affection and follicular psorospermosis.

M. Darier concludes by stating his reasons for preferring the name he has adopted to that of acanthosis nigricans, to which even papillomatosis nigricans would even be preferable.—*British Journal of Dermatology, December*, 1893.

---

### NOTES ON SKIN DISEASES.

Dr. W. F. Breakey, of the University of Michigan, Ann Arbor, has some notes on the cases seen in his clinic for skin diseases, in the January, 1894, *Journal of Cutaneous and Genito-Urinary Diseases.*

He found many cases in which more than one skin disease existed; as eczema with acne, with seborrhea, with psoriasis. Often a combination with "parasitic disease" and syphilis.

Many skin diseases existed along with constitutional disease, necessitating general medication as well as local. [General medication in the majority of skin diseases consists in treating the constitutional disorder without regard to the skin disease, just as it would be treated were there no skin disease.—ED.]

Psoriasis was often followed by rheumatism or gout ("in its wake," he says), and was relieved or cured by appropriate treatment for the general trouble.

Tonics and cod liver oil useful in many cases of eczema, both in infants and adults.

Chronic cases of acne and seborrhea did better as a rule with saline laxatives, care in diet, sufficient exercise and puncturing the (acne) pustules and indurate lesions.

Gives the history of a keloid tumor about the size of a half dollar on the site of a boil on the sternum ten years before. Removal of the keloid was followed by return in a year. The doctor then removed the growth, making a wide elliptical incision. The skin was loosened so as to allow coaptation. Interrupted sutures, supported by long adhesive strips. Union by first intention. No recurrence at last report.

Early falling of the hair he is inclined to attribute to too much barber shop.

---

### TREATMENT OF KELOID WITH SUBCUTANEOUS INJECTIONS OF CREOSOTE OIL.

P. Marie, *Journ. de Mal. Cut. et Syph.*, 1893, *p.* 162, recommends injections of twenty per cent. creosote oil into the keloid growth. Will destroy the growth without fear of any bad results This treatment probably available in other diseases, as cancer and lupus.—*Monatschefte für Praktische Dermatologie*, xvii., *No.* 11.

[A properly insulated electrolytic needle, with sufficient current, might effect as good results in keloid. Electrolysis or creosote injections rather risky in cancerous growths, while destroying part might stimulate the remainder.—ED.]

---

### ETIOLOGY AND HISTOLOGY OF CHALAZION.

R. Krause has investigated this subject. After microscopic examination of nine cases he concluded it was not a local tubercular disease, but a chronic, inflammatory process of the meibomian glands, beginning in the surrounding connective tissue. Various micro-organisms considered causative, entering through the lymphatics of the peri-acinous tissue. Disease predisposed to by "scrofulous" blepharo-conjunctivitis.—*Monatschefte für Praktische Dermatologie*, xvii., *No.* 11.

### TREATMENT OF ACCUMINATE GENITAL WARTS WITH PURE CARBOLIC ACID.

Derville, *Semaine Med.*, 1893, *No.* 35, first covers adjacent parts with vaseline, then applies carbolic acid liquefied by warming. Very slight pain. Repeat when eschar has separated. Result, certain and early recovery.—*Monatschefte für Praktische Dermatologie*, xvii., *No.* 11.

---

### RIGIDITY OF OS FROM SYPHILIS.

Crouzat, Pinard and Maygrier reported to the French Obstetrical Society cases of rigidity of the "neck of the uterus," result of chancrous scars or tertiary sclerosis.—*Monatschefte für Praktische Dermatologie*, xvii., *No.* 11.

